# Resolution of Keratoacanthoma Type Squamous Cell Carcinoma Following Intralesional Therapy With Methotrexate

**DOI:** 10.7759/cureus.8092

**Published:** 2020-05-13

**Authors:** Andrea Sisti, Maria T Huayllani, Daniel Boczar, Scott Fosko, Antonio J Forte

**Affiliations:** 1 Plastic Surgery, Cleveland Clinic Ohio, Cleveland, USA; 2 Plastic Surgery, Mayo Clinic Florida, Jacksonville, USA; 3 Dermatology, University of Florida, Gainesville, USA

**Keywords:** keratoacanthoma, squamous cells carcinoma, methotrexate

## Abstract

Keratoacanthoma is considered a variant of squamous cell carcinoma prone to spontaneous involution, but it may also rapidly grow and invade surrounding tissues. Herein, we report a case of keratoacanthoma-type squamous cell carcinoma that resolved after intralesional therapy with methotrexate.

## Introduction

Keratoacanthoma is considered a less-aggressive subtype of cutaneous squamous cell carcinoma, but occasionally behaves more aggressively and invades surrounding tissue. It usually occurs on sun-exposed sites in the older population and its classification is still debated in the literature [1]. We present a case report of a keratoacanthoma-type squamous cell carcinoma treated with an intralesional therapy of methotrexate.

## Case presentation

A 95-year-old man presented for a tender lesion on the right thumb that had been present for more than two weeks. His medical history included numerous nonmelanoma skin cancers, no family history of melanoma, no other skin lesions or rashes of concern, and no associated unexplained fevers or weight loss. He appeared well developed, well nourished, and in no apparent distress.

**Figure 1:**
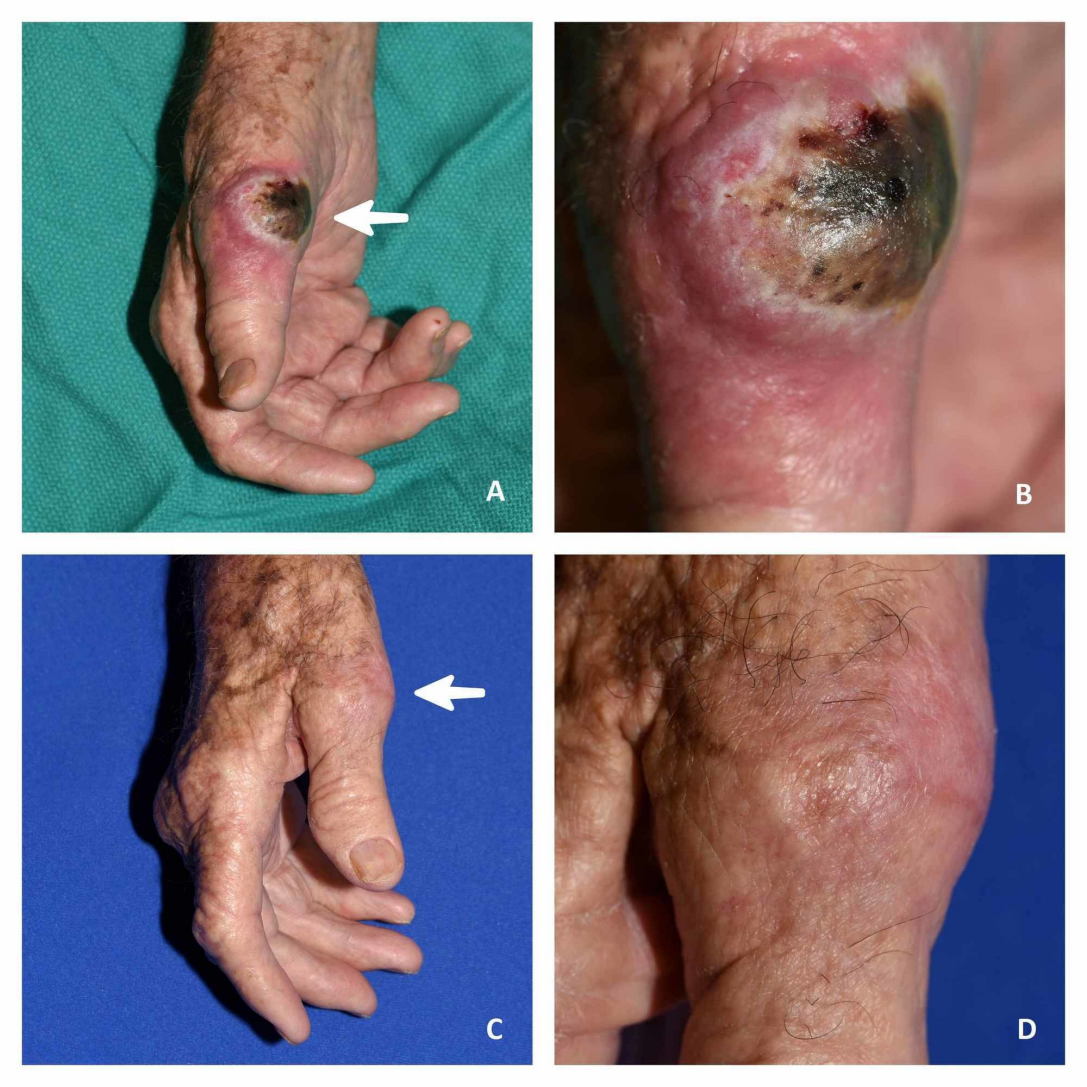
Pre- and post-treatment pictures. (A, B) Pre-treatment pictures. The arrow shows lesion in the first digit of the right hand. (C, D) After three methotrexate injections (three months after the first methotrexate intralesional treatment). The arrow shows the lesion completely disappeared.

On the palmar aspect of the right thumb metacarpophalangeal joint was a 4.5×3.5 cm keratotic indurated flesh-colored nodule with a central keratotic core (Figure 1). It did not appear to be mobile, but did have a suggestion of deep extension. It was tender to palpation, with moderate pain severity, and could not be drained. Examination of the right epitrochlear and right axillary lymph node region was unremarkable.

**Figure 2:**
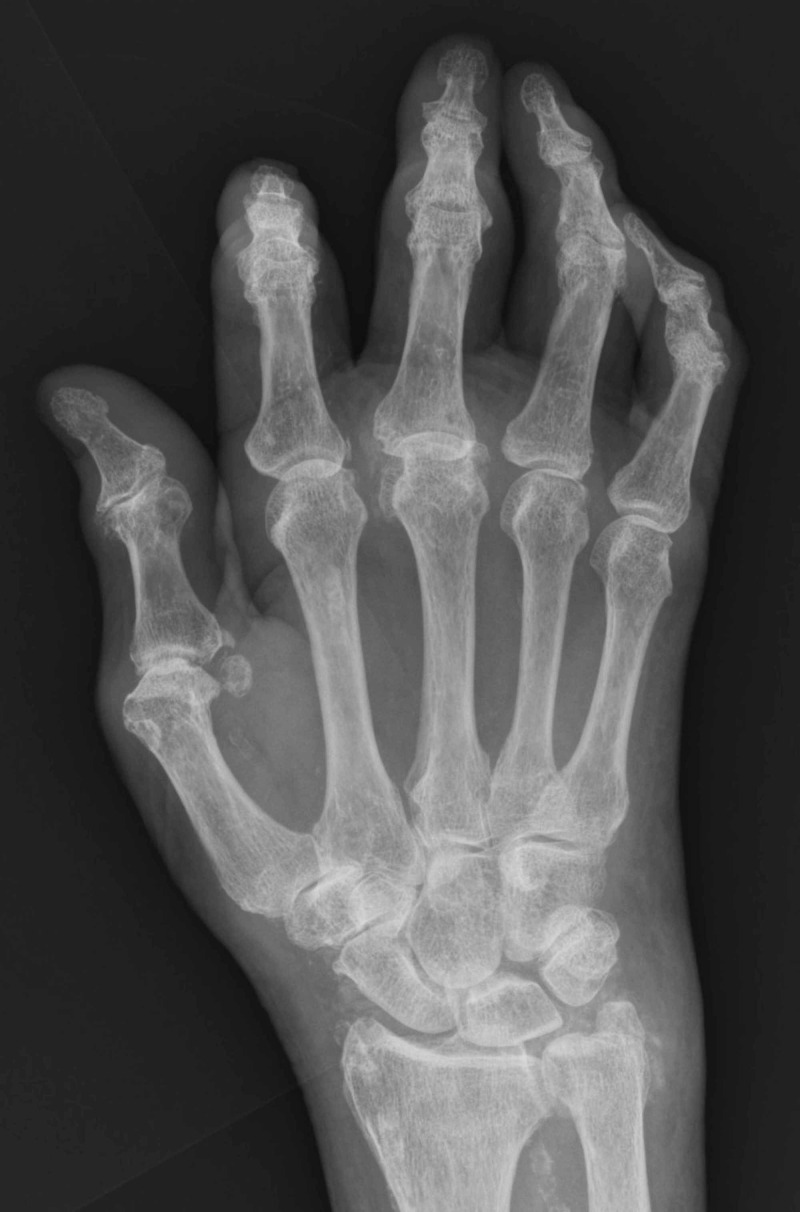
Radiography of the right hand. No bone involvement is observed.

Radiography of the right hand excluded bone involvement (Figure 2). A 3-mm punch biopsy was performed and showed well-differentiated squamous cell carcinoma with regressing keratoacanthoma-type features, with deep biopsy edge involved. 

The patient and his son declined the proposed treatment with amputation due to his age. They accepted the proposed nonsurgical treatment with intralesional methotrexate. After an alcohol and Techni-Care skin preparation using an antiseptic from the Care-Tech Laboratories, Inc. (St. Louis, MO), the tumor borders at four quadrants and central portion were injected with 12.5 mg/mL each of intralesional methotrexate, for a total of 2 mL, 25 mg of methotrexate. The treatment was repeated twice more at an interval of one month.

The tumor showed considerable regression after the first injection, and had completely disappeared at three-month follow-up. The patient tolerated the procedure without any difficulty or adverse effect.

## Discussion

The standard treatment of a keratoacanthoma is the surgical excision of the lesion, but several nonsurgical treatments, including radiation therapy, oral retinoids, and intralesional application of 5-fluorouracil, methotrexate, and interferon α-2a, have been reported for patients who refuse or are unable to tolerate surgery [2].

Methotrexate is a folate antagonist that binds and inhibits dihydrofolate reductase and thymidylate synthase in the folate cycle and the pathway of de novo biosynthesis of purine nucleotides from ribose 5-phosphate, thus inhibiting DNA synthesis in actively dividing cells [3]. It is commonly used in systemic chemotherapy, but may be placed directly into neoplasm, with less systemic toxicity.

Previous studies showed a high success rate of intralesional methotrexate for the treatment of keratoacanthoma [4]. Intralesional methotrexate has also been used for the treatment of nail psoriasis, basal cell carcinoma, nodular cutaneous amyloidosis, and pyoderma gangrenosum, but it has been ineffective in the treatment of cutaneous metastases of malignant peripheral nerve sheath tumors [5,6]. Methotrexate is relatively inexpensive and should be taken into consideration when choosing an intralesional agent for keratoacanthoma-type squamous cell carcinoma.

## Conclusions

Intralesional therapy with methotrexate is a potential noninvasive medical treatment that should be considered for all keratoacathoma-type squamous cell carcinomas when patients are not amenable to surgery or radiation therapy. It is an inexpensive and relatively painless procedure that offers a great aesthetic outcome and might prevent a wide surgical resection and further reconstruction.
